# Functional characterization and related evolutionary implications of invertebrate gonadotropin-releasing hormone/corazonin in a well-established model species

**DOI:** 10.1038/s41598-021-89614-5

**Published:** 2021-05-11

**Authors:** István Fodor, Réka Svigruha, Zsolt Bozsó, Gábor K. Tóth, Tomohiro Osugi, Tatsuya Yamamoto, Honoo Satake, Zsolt Pirger

**Affiliations:** 1 NAP Adaptive Neuroethology, Balaton Limnological Research Institute, Eötvös Loránd Research Network (ELKH), Klebelsberg Kuno u. 3., Tihany, 8237 Hungary; 2 Department of Medical Chemistry, University of Szeged, Szeged, Hungary; 3 Bioorganic Research Institute, Suntory Foundation for Life Sciences, 8-1-1 Seikadai, Seika, Souraku, Kyoto 619-0284 Japan

**Keywords:** Neuroscience, Endocrinology

## Abstract

In vertebrates, gonadotropin-releasing hormone (GnRH) peptide is the central mediator of reproduction. Homologous peptides have previously also been identified in molluscan species. However, emerging evidence suggests that these molecules might serve diverse regulatory functions and proposes to consider them as corazonin (CRZ). We previously isolated the full-length cDNA of the invGnRH/CRZ peptide (termed ly-GnRH/CRZ) in the well-established invertebrate model species, the great pond snail *Lymnaea stagnalis*; however, its predicted functions remain to be verified. In this study, we first confirmed the presence of the deduced active peptide from the central nervous system of *L. stagnalis*. Further, we performed in vivo and in vitro studies to explore the functions of ly-GnRH/CRZ. Injection of sexually mature specimens with synthetic active peptide had an inhibitory effect on locomotion and an acceleratory effect on egg-laying, but had no effect on feeding. The previously predicted modulatory effect of ly-GnRH/CRZ was supported by its identified co-localization with serotonin on the surface of the heart atria. Lastly, we demonstrated not only the presence of ly-GnRH/CRZ in the penial complex but also that ly-GnRH/CRZ-containing neurons project to the efferent penis nerve, suggesting ly-GnRH/CRZ may directly modulate the motor output of this peripheral tissue. Overall, our findings strongly support that ly-GnRH/CRZ is a multifunctional neuropeptide. These results contribute to the understanding of the GnRH superfamily and, more broadly, disciplines such as comparative endocrinology and neurobiology.

## Introduction

In the last years, the interpretation of the functions of the gonadotropin-releasing hormone (GnRH) neuropeptide superfamily has changed tremendously. One of the main drivers of this conceptual change is investigating invertebrate GnRH/corazonin (invGnRH/CRZ) peptides, initially termed invGnRH. The original nomenclature of these peptides has led to some confusion regarding their evolutionary lineage and function since the term invGnRH indicates a highly specialized reproductive function as seen in vertebrate GnRH^1^. However, emerging evidence suggests that invGnRH/CRZ peptides are likely to assume diverse biological effects: not only reproductive, but also motor, behavioral, and neuromodulatory functions^2–7^. CRZs, first discovered in the American cockroach (*Periplaneta americana*)^8^, are multifunctional peptides and part of the GnRH superfamily^1^. The misinterpretation of sequence and function of the first discovered invGnRH/CRZ (in the common octopus, *Octopus vulgaris*) has led to the its name oct-GnRH^2,9^; however, recent structural, phylogenetic, and phylogenomic analyzes suggest that molluscan GnRHs are more related to CRZs and a nomenclature change should be considered^1,5,10–13^. A more global understanding of invGnRH/CRZ evolution requires further exploration of species-specific functions and structure of invGnRH/CRZ molecules.

Although the complete sequences of invGnRH/CRZ peptides have been identified in some molluscan species (Supplementary Table 1), the functions of these molecules were investigated in *O. vulgaris* and the sea hare (*Aplysia californica*) in the most detail (both reproductive and non-reproductive functions). Anatomical characterization of oct-GnRH/CRZ revealed that the transcript and peptide are present in the central nervous system (CNS), auricle of the heart, and peripheral reproductive organs^2^. In the CNS, the detected oct-GnRH/CRZ-immunopositive neurons, neuronal clusters, and fibers are responsible for feeding, arm movement, heart control, and memory processing^2^. Functional studies showed that oct-GnRH/CRZ stimulates steroidogenesis from gonads^9^, induces the contraction of oviducts, and has ionotropic and chronotropic effects on the heart^2^. Moreover, oct-GnRH/CRZ was suggested to be involved in a series of egg-laying behaviors^3^. In *A. californica*, ap-GnRH/CRZ was only found in CNS; the transcript and peptide distribution implied its functions in feeding, motor control of foot and parapodia, and interganglionic neuromodulation^6^. Furthermore, functional studies confirmed the regulatory role in feeding and motor activity^4,14^. Despite these findings, knowledge of the functions of invGnRH/CRZ peptides is still incomplete. This prevents our understanding of how the GnRH superfamily has evolved functionally over time.

We have recently determined the cDNA sequence of invGnRH/CRZ (termed ly-GnRH/CRZ) in *L. stagnalis* and characterized the transcript and peptide distribution in the CNS and some peripheral organs^5^. The functional/active peptide was deduced to be an undecapeptide with an N-terminal pyroglutamate and a C-terminal alanine amide (pQNYHFSNGWYA-NH_2_). Based on the anatomical distribution, we predicted that, coinciding with the functions mentioned above, ly-GnRH/CRZ might regulate feeding, locomotion, heart control, and reproduction. However, these functions have not yet been confirmed. Therefore, the goal of the present study was to functionally characterize the ly-GnRH/CRZ peptide. Since *L. stagnalis* has been a widely used model organism in invertebrate neuroscience for decades^10,15–19^, the investigation of ly-GnRH/CRZ could contribute not only specifically to the understanding of the GnRH superfamily but also more broadly to disciplines such as comparative endocrinology and neurobiology. This species possesses unique features such as a well-defined CNS, clearly defined behaviors, and accessible anatomy that can facilitate the characterization of ly-GnRH/CRZ. To accomplish our goal, we first verified the presence of the deduced active peptide in the CNS of *L. stagnalis* by mass spectrometry (MS) analysis. Next, we performed behavioral assays where we injected synthetic active peptide into sexually mature specimens of *L. stagnalis*. In addition, we utilized immunohistochemistry (IHC) and retrograde labeling to explore further functions of ly-GnRH/CRZ. Our findings support the role of ly-GnRH/CRZ as a neuropeptide with diverse functions.

## Results

### Peptide identification of ly-GnRH/CRZ

**Figure 1:**
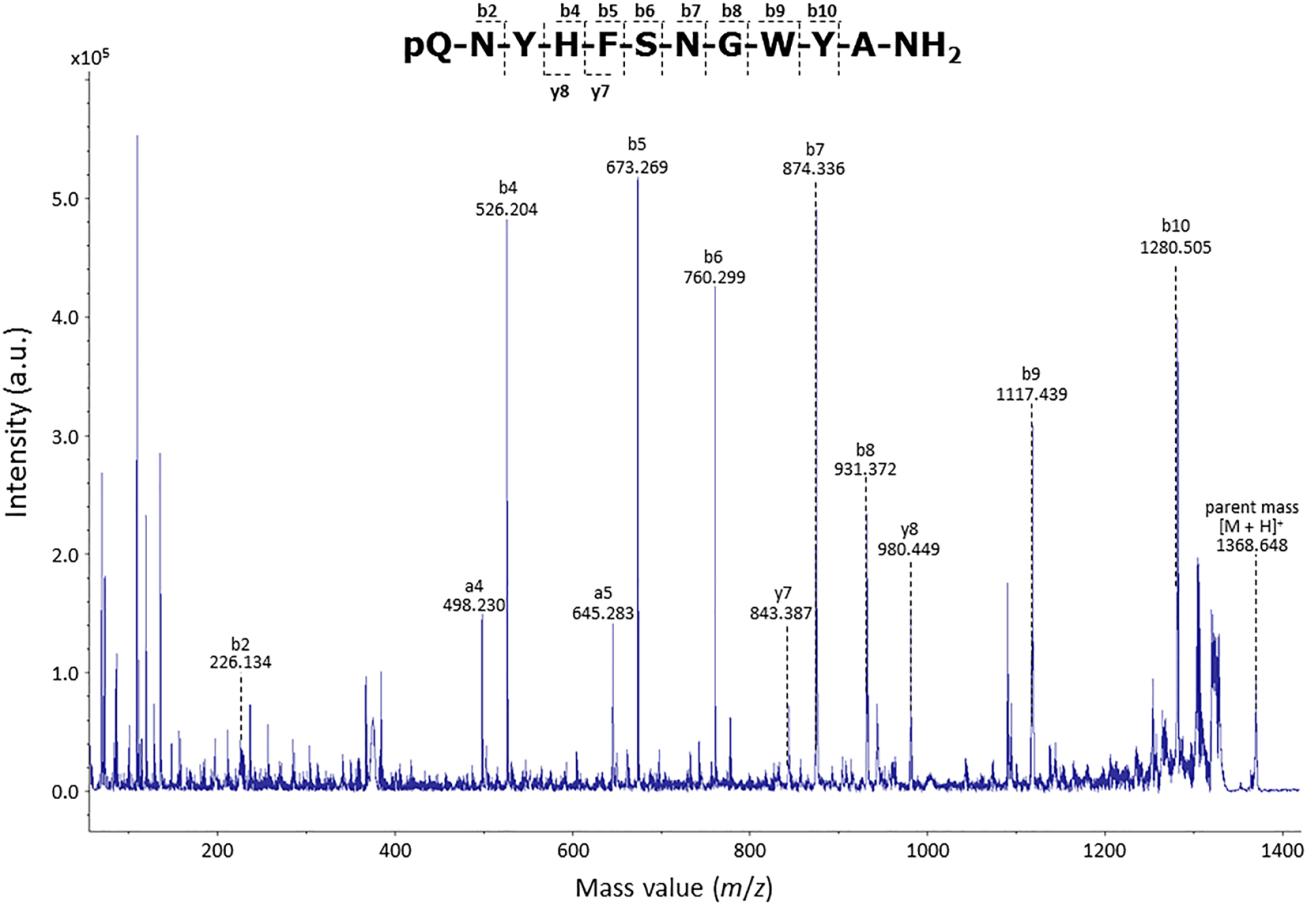
Identification of ly-GnRH/CRZ by mass spectrometry. MALDI-TOF/TOF MS/MS spectrum of ly-GnRH/CRZ. Parent ion [M + H]^+^, b-ions, y-ions, and a-ions of ly-GnRH/CRZ are labeled.

MS analysis of a fraction from the CNS corresponding to the elution times of 31–32 min allowed the detection of a single peak corresponding to an MS value of 1368.648 [M + H]^+^ for the active ly-GnRH/CRZ peptide. Subsequent MALDI-TOF/TOF MS/MS analysis revealed that the peptide sequence was pQNYHFSNGWYA-NH_2_, as depicted in Fig. 1. These results confirmed that the peptide sequence was compatible with the deduced pre-pro ly-GnRH/CRZ protein sequence (#QIH29241) published previously^5^.

### ly-GnRH/CRZ had no effect on feeding

**Figure 2:**
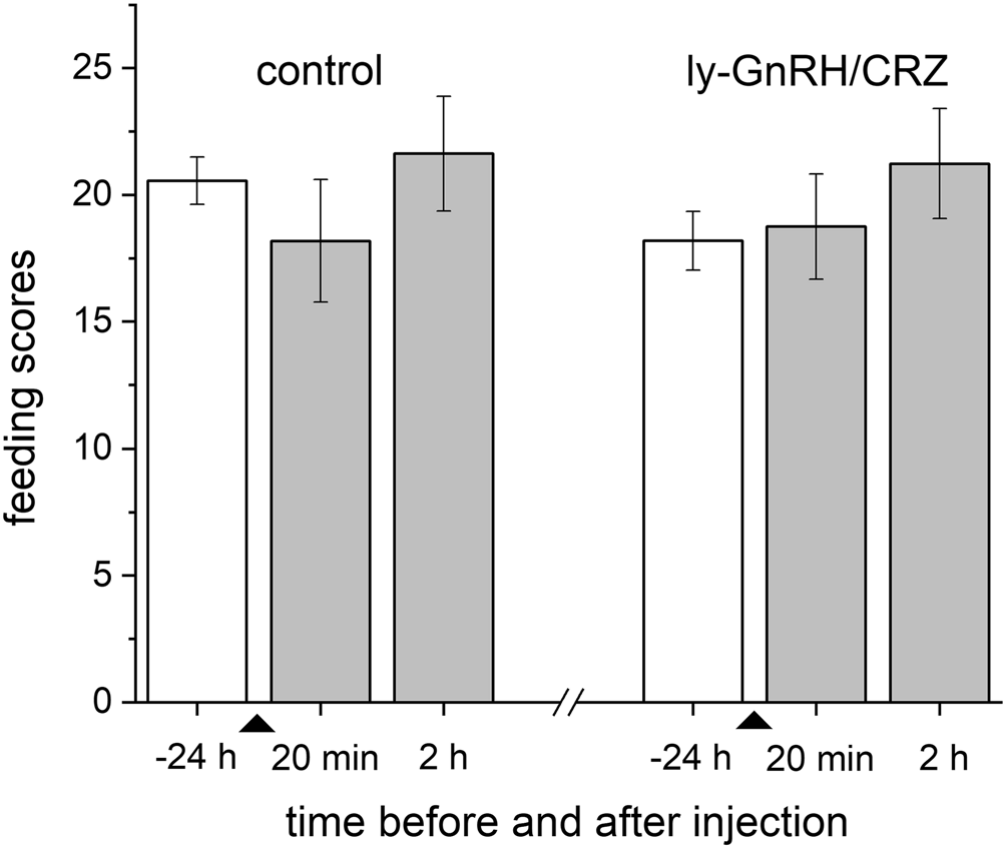
Feeding activity (number of rasps/2 min) of experimental groups. White and grey columns represent the score before and after injection, respectively. Each bar represents mean ± SEM (n = 10 animals/group/replicates). Triangles indicate the time point of peptide injection. No significant differences were observed between the control and ly-GnRH/CRZ injected groups.

ly-GnRH/CRZ peptide injection (Supplementary Fig. 3) could not alter the feeding activity. Compared to the control, no significant differences were detected in the number of bites in the treated group at 20 min or 2 h after the injection (Fig. 2).

### ly-GnRH/CRZ acutely decreased locomotor activity

**Figure 3:**
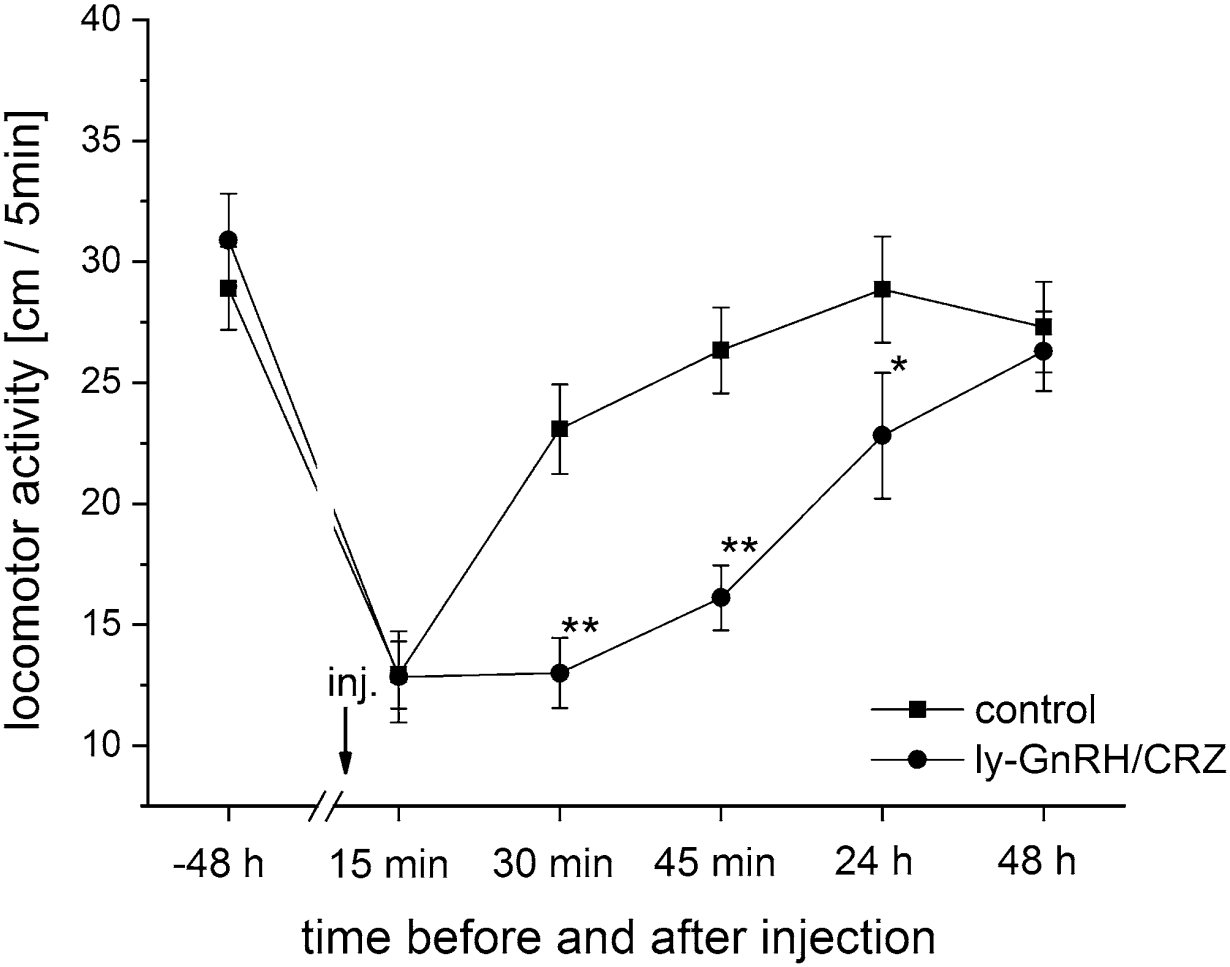
Locomotor activity (distances covered by snails/5 min) of experimental groups. Each data point represents mean ± SEM (n = 10 animals/group/replicates). Within a single time point, significance of differences to the control is indicated by asterisks (*P < 0.05 and **P < 0.01).

Figure 3 presents the effect of ly-GnRH/CRZ injection (Supplementary Fig. 4) on locomotor activity. A two-way repeated-measures ANOVA revealed significant effects of time (observation points) [F(5, 251) = 20.03, *P* < 0.005] and treatment [F(1, 251) = 7.96, *P* < 0.001], and a significant time × treatment interaction [F(11, 251) = 3.90, *P* < 0.005]. Further analysis with a two sample t-test revealed that animals injected with synthetic ly-GnRH/CRZ started to exhibit significantly decreased (*P* < 0.01) locomotor activity within 25 min after injection (Fig. 3). This effect was also observed even at 24 h after injection (*P* < 0.05). The effect was no longer observed at 48 h post-injection (Fig. 3).

### ly-GnRH/CRZ affected egg-laying behavior

ly-GnRH/CRZ peptide injection (Supplementary Fig. 5) significantly influenced egg-laying behavior (Table 1). The number of normal egg masses laid by the injected group (29.66 ± 1.33, *P* < 0.001) was significantly higher than the control group (7.66 ± 0.33). In this way, the number of normal laid eggs in the injected group (1175 ± 49.65, *P* < 0.001) also increased significantly comparing to the control (533 ± 19.05). Simultaneously, the average egg number/egg mass significantly decreased in the injected group. Furthermore, the egg masses of the injected group showed a significant qualitative decline manifested in the increased number of polyembryonic egg masses (7.66 ± 1.2, *P* < 0.01) compared to the control (0.33 ± 0.33).

**Table 1:** Egg laying behavior of experimental groups.

	Control	ly-GnRH/CRZ injected
Number of normal egg masses	7.66 ± 0.33	29.66 ± 1.33***
Number of polyembryonic egg masses	0.33 ± 0.33	7.66 ± 1.2**
Number of normal eggs	533 ± 19.05	1175 ± 49.65***
Number of polyembryonic eggs	1.33 ± 1.33	287 ± 11.93***
Average egg number/egg mass	69.82 ± 1.59	39.38 ± 2.29***

Values represent mean ± SEM (n = 10 animals/group/replicates); significant results, in comparison with the control, are indicated by asterisks (**P < 0.01 and ***P < 0.001).

### Localization of ly-GnRH/CRZ in central and peripheral tissues

**Figure 4:**
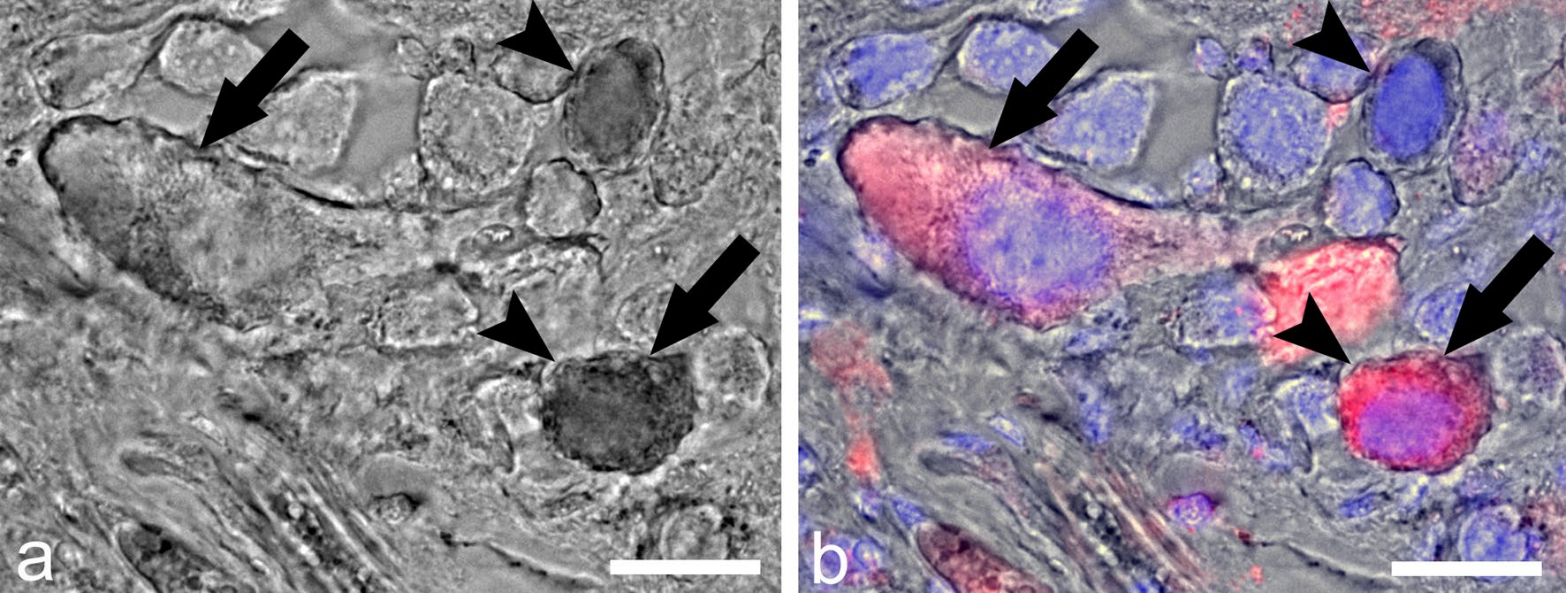
Double-labeling of ly-GnRH/CRZ neurons in the anterior lobe of the right cerebral ganglion by IHC and nickel-lysine backfill. (**a**,**b**) represent the same section. The section after nickel backfill was visualized without (**a**) and with (**b**) IHC imaging. Red color: ly-GnRH/CRZ immunostaining; blue color: nuclei staining. Arrow represents a ly-GnRH/CRZ-ir neuron. Arrowhead indicates a backfilled neuron. The double-labeled neuron is labeled by both arrow and arrowhead. Scale bar = 25 µm.

To determine if ly-GnRH/CRZ-containing neurons project to the penis nerve responsible for the motor innervation of the penial complex, CNS neurons were labeled via the penis nerve with retrograde tracing followed by ly-GnRH/CRZ IHC. All relevant neurons and neuronal clusters/regions, such as the pedal Ib cluster of the right pedal ganglion, known from the literature to project to the penis nerve^17,20^ were stained in the given ganglia of the CNS (Supplementary Fig. 6). We observed double-labeled neurons in the anterior lobe but not in the ventral lobe of the right cerebral ganglion. In Fig. 4, one double-labeled neuron can be observed in the representative section of the anterior lobe. Some ly-GnRH/CRZ-immunoreactive (ir) neurons not labeled with nickel chloride were also observed suggesting that not all ly-GnRH/CRZ-containing neurons project to the penis nerve. The positions of the ly-GnRH/CRZ-ir neurons were consistent with the positions previously reported for this ganglion^5^.

**Figure 5:**
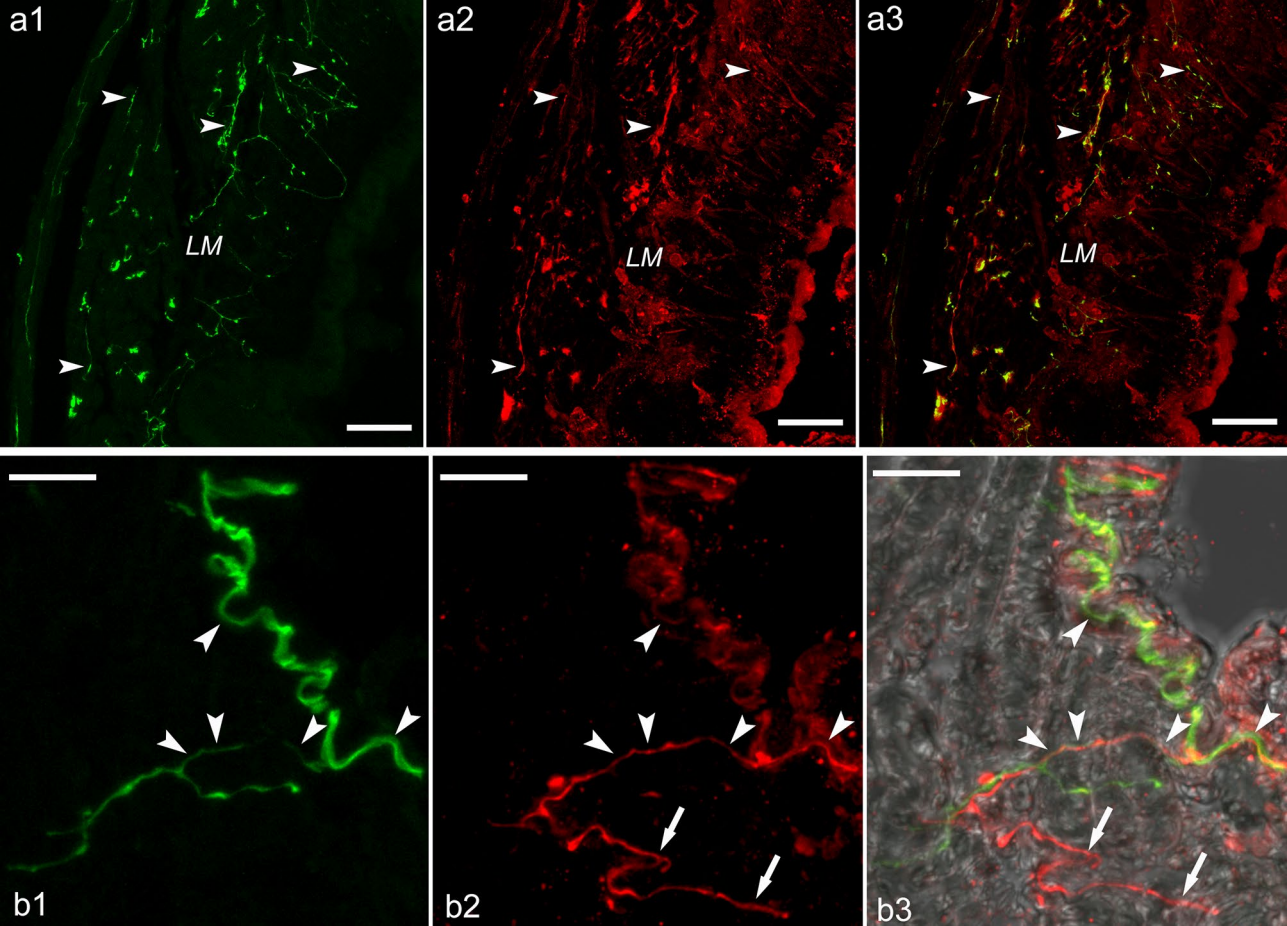
Representative 5-HT (green) and ly-GnRH/CRZ (red) IHC of the preputium (**a1**–**a3**) and the heart atria (**b1**–**b3**). Axon fiber bundles containing both 5-HT and ly-GnRH/CRZ (arrowheads) can be seen on the longitudinal muscle of the preputium. On the surface of the heart atria muscle, axon fiber bundles containing only ly-GnRH/CRZ (arrows) or both 5-HT and ly-GnRH/CRZ (arrowheads) are present. Scale bars = 40 µm (**a1**–**a3**) and 10 µm (**b1**–**b3**). *LM* longitudinal muscle.

Among peripheral tissues, we investigated the penial complex (Supplementary Fig. 7) and heart by confocal imaging to reveal if ly-GnRH/CRZ modulates the multi-messenger innervation of these tissues as predicted previously^5^. Our IHC revealed neuronal elements containing both ly-GnRH/CRZ and 5-HT in the longitudinal muscle of the preputium (Fig. 5a1–a3). Besides the preputium, ly-GnRH/CRZ-ir and 5-HT + ly-GnRH/CRZ-ir axon fibers are present on the surface of the heart atria muscle fibers; however, the heart muscle fibers themselves were not immunopositive (Fig. 5b1–b3).

## Discussion

To the best of our knowledge, among mollusks, invGnRH/CRZ has so far been verified by MS analysis only in the cephalopod *O. vulgaris*^21^, two marine shellfish, the Pacific oyster *Crassostrea gigas*^22^, Yesso scallop *Mizuhopecten yessoensis*^23^, and the pacific abalone *Haliotis discus hannai*^24^. Hence, this is the first study to present the MS identification of an invGnRH/CRZ in a freshwater gastropod mollusk. The previous MS analyses demonstrated that these bivalves have two forms of invGnRH/CRZs: an amidated undecapeptide and a non-amidated dodecapeptide^22,23^. In contrast, MS investigation of *O. vulgaris* revealed only the presence of an amidated dodecapeptide^21^. In our previous study, we supposed that, similar to bivalves, both amidated undecapeptide (pQNYHFSNGWYA-NH_2_) and non-amidated dodecapeptide (pQNYHFSNGWYAG-OH) forms may be present in the CNS of *L. stagnalis*^5^. However, the MS analysis of the present study yielded only the presence of the amidated undecapeptide form. In vivo administration of the oyster amidated undecapeptide was demonstrated to induce spawning in the Sydney rock oyster *Saccostrea glomerata*^25^, although differences in bioactivities of the two forms were not investigated. In *Mizuhopecten yessoensis*, the amidated undecapeptide was found to stimulate spermatogonial cell proliferation and masculinization^26–28^. In *H. discus hannai*, injection of the amidated undecapeptide induced spawning and ovulation^24^. In *A. californica*, the effects of both forms were investigated, but only the amidated undecapeptide was found to be biologically active^4^. These findings and our results suggest that utilization of the amidated undecapeptide as the functional invGnRH/CRZ molecule is a common feature in bivalves and gastropods. However, the evolutionary significance of the unusual amidated dodecapeptide as the functional molecule in *O. vulgaris* (and in cephalopods in general) remains to be understood.

Previous functional studies in *O. vulgaris* and *A. californica* demonstrated that invGnRH/CRZ peptides are involved in diverse functions, including feeding, locomotion, heart control, interganglionic neuromodulation, and reproduction^2,4,6,9^. In our previous study, we predicted that ly-GnRH/CRZ might also serve as a multifunctional peptide^5^. Even though the B3 and B4 motoneurons of buccal ganglia (responsible for the execution of feeding^29^) and CV cells of cerebral ganglia (linked to feeding control via the lip motoneurons^30^) showed ly-GnRH/CRZ-immunopositivity suggesting a possible role of ly-GnRH/CRZ in feeding^5^, we did not detect any acute effect on feeding activity after the peptide injection (Fig. 2). This contrasts with findings that ap-GnRH/CRZ injection acutely inhibited feeding in *A. californica* ^4^. Because of the ly-GnRH/CRZ-immunoreactivity of A cluster neurons of the pedal ganglia (involved in control of foot cilia^31^), we also predicted a possible function in locomotion^5^. This was confirmed in this study since animals after injection with ly-GnRH/CRZ peptide exhibited a significantly decreased locomotor activity (Fig. 3). Based on this and previous findings in *A. californica* and *O. vulgaris*, the involvement of invGnRH/CRZ in locomotor activity seems to be universal in the molluscan species investigated. This is supported by the suggestion that invGnRH/CRZ is involved in the foot locomotion of *M. yessoensis*^23^*.* Egg-laying regulatory caudodorsal cells (CDCs;^32^) of cerebral ganglia also showed ly-GnRH/CRZ-immunopositivity indicating that ly-GnRH/CRZ may have a potential role in egg-laying^5^. To note, this regulatory role of GnRH/CRZ has already been suggested by previous studies in *L. stagnalis* and the ass's-ear abalone *Haliotis asinina*^17,33^. In the present study, specimens after injection with ly-GnRH/CRZ showed a significantly changed egg-laying behavior manifested in the increased number of laid egg masses and eggs (Table 1). This result contrasts with findings that injection of ap-GnRH/CRZ did not affect the egg-laying behavior of *A. californica* ^4^. However, *A. californica* bag cells, which are homologous to CDCs and responsible for egg-laying^34^, did not show ap-GnRH/CRZ-immunopositivity^6,35^. Interestingly, ly-GnRH/CRZ also affected the frequency of polyembryonic eggs (Table 1), but this phenomenon remains to be understood. In our previous study, we detected ly-GnRH/CRZ-immunopositive neurons in the ventral- and anterior lobe of the right cerebral ganglion known to innervate the penial complex^5^. The uptake of nickel chloride by some ly-GnRH/CRZ-immunopositive neurons in the anterior lobe from the penis nerve supports the direct projection of ly-GnRH/CRZ neurons to this efferent nerve (Fig. 4). The regulation of the penial complex is basically implemented by synchronization of numerous neurotransmitters, such as conopressin, FMRFamide, and 5-HT, arriving from the CNS via the penis nerve^17,36^. Our IHC revealed neuronal elements containing both ly-GnRH/CRZ and 5-HT in the longitudinal muscle of the preputium (Fig. 5a3). Given the presence of ly-GnRH/CRZ in the penial complex and that ly-GnRH/CRZ-containing neurons project to the penis nerve, ly-GnRH/CRZ is likely to directly modulate the motor output of this peripheral tissue. Taking all of our findings on reproduction into consideration, we expect that ly-GnRH/CRZ is involved in male mating behavior, mainly in insemination. It is worth mentioning that other studies performed in insects have reported that CRZ signaling is required for sperm transfer and copulation duration^37,38^. As mentioned above, another molluscan studies focusing on reproduction demonstrated that invGnRH/CRZ stimulates spermatogonial cell proliferation and masculinization but inhibits oocyte growth in *M. yessoensis*^26,28^, induces oogonia and oocyte proliferation in *H. asinina*^39^, and enhances ovulation and spermatogonial- and oogonial cell proliferation in *H. discus hannai*^24,40^. We conclude that, without being a universal reproduction-mediating peptide, invGnRH/CRZ is widely utilized in mollusk reproduction. Besides, the involvement of invGnRH/CRZs in reproduction seems to be highly species-specific. Lastly, because of the presence of ly-GnRH/CRZ-immunopositive fibers on the surface of heart atria muscles, ly-GnRH/CRZ-immunoreactivity of RPeD1 cell of the right pedal ganglion, and ly-GnRH/CRZ-immunopositivity in HIJK cells of the visceral ganglion (involved in heart control^41^), we predicted that ly-GnRH/CRZ might also contribute to the regulation of the heart^5^, which is known to be modulated by a rich cocktail of chemicals^16,41^. This is supported by the co-localization of ly-GnRH/CRZ and 5-HT, the classic transmitter of excitatory heart motoneurons in *L. stagnalis*^41,42^, in the neuronal element on heart atria muscles (Fig. 5b3). This potential modulatory effect coincides with previous findings in *O. vulgaris*^2,21^, but further investigations (e.g., in vivo pharmacological assay on isolated heart preparation) are required to further validate this function.

In summary, we confirmed the presence of the deduced active peptide in the CNS and could verify a regulatory role in locomotion and reproduction from the earlier predicted functions. Our results further suggest that the amidated undecapeptide is utilized in bivalves and gastropods. The potential regulatory role of ly-GnRH/CRZ in heart control requires further physiological investigations. Interestingly, we did not find any link to the feeding. It should be noted that we used only one concentration (10 µg peptide/each animal) during the injection so it remains possible that this concentration was too low to cause any effect on feeding. Therefore, future physiological works should aim to use further peptide concentrations. The mode of action of ly-GnRH/CRZ seems to be implemented via direct stimulation and neuromodulation, and this has previously been concluded with arthropod CRZs as well^43^. Although there is still only a limited number of functional studies with invGnRH/CRZs, our findings add to a growing body of literature supporting invGnRH/CRZ peptides as being responsible for the regulation of both reproductive and non-reproductive functions. To note, most of the identified functions of invGnRH/CRZ peptides show species-specificity hence it is difficult to find a common denominator. Although the involvement in locomotor activity seems to be universal, additional functional studies and suitable protostomian models are required to further explore the evolution of the GnRH superfamily.

## Materials and methods

### Experimental animals

For this study, *L. stagnalis* specimens were obtained from our laboratory-bred stocks. Snails were kept in large holding tanks (with a 100 individuals/tank stocking density) containing 10 L oxygenated artificial snail water with low copper content at a constant temperature of 20 °C (± 1 °C) on a light:dark regime of 12 h:12 h. Specimens were fed on lettuce ad libitum three times a week. All animals used in the experiment originated from the same breeding cohort, and were thus all of the same age (five months old, mature snails). All procedures were performed according to the protocols approved by the Scientific Committee of Animal Experimentation of the Balaton Limnological Institute (VE-I-001/01890-10/2013).

### Peptide extraction, purification, and MS analysis

For ly-GnRH/CRZ extraction, the whole CNS was dissected from the animals (n = 35). The CNSs were pooled and extracted following a previously published protocol^23^. The tissues were pulverized by grinding under liquid nitrogen and boiled for 7 min in 10 mL of distilled water. After cooling down, acetic acid was added at a final concentration of 5%. The solution was homogenized with a Polytron PT 1200 homogenizer (Kinematica AG) for 1–2 min (intermittently) at 4 °C. The homogenate was centrifuged at 10,000×*g* for 30 min at 4 °C, and the resulting precipitate was again homogenized (10 mL distilled water with 5% acetic acid) and centrifuged. The supernatants were pooled and lyophilized. The sample was reconstituted with 1 mL 0.1% trifluoroacetic acid (TFA)-containing water induced by ultrasonic and vortex mixing for solid-phase extraction. The procedure was as follows: a disposable C18 cartridge (100 mg/1 mL, #52602-U, Sigma-Aldrich) was conditioned with 0.1% TFA-containing 60% acetonitrile (ACN; 3 mL) and equilibrated with 0.1% TFA-containing water (2 mL), then the sample was passed through the cartridge followed by washing with 0.1%-TFA containing water (2 mL), finally the retained materials were eluted with 0.1% TFA-containing 60% ACN (1 mL). The eluted samples were further subjected to a high-performance liquid chromatography (HPLC) column (ODS-80Ts 4.6 mm I.D. × 150 mm; Tosoh, Tokyo, Japan) on an HPLC system (Shimadzu Prominence-i LC-2030C) with a linear gradient of 4.5–49.5% ACN containing 0.1% TFA for 50 min at a flow rate of 1 mL/min, and the eluted fractions were collected every 1 min (Supplementary Fig. 1). Each fraction was analyzed by matrix-assisted laser desorption/ionization time-of-flight/time-of-flight MS (MALDI-TOF/TOF MS) (rapifleX; Bruker Daltonics, Bremen, Germany).

### Peptide synthesis and injection

The synthesis of active ly-GnRH/CRZ peptide was performed using a solid-phase procedure with Fmoc-chemistry. Peptide chains were elongated on a Tentagel S-Ram resin (0.23 mmol/g) and the synthesis was performed using a CEM Liberty Blue machine. The peptide was detached from the resin by using a mixture containing 90% TFA, 4% water, 2% dithiothreitol (DTT), 2% triisopropylsilane (TIS), and 2% p-cresol. The resulted crude peptide was purified by reverse-phase HPLC (RP-HPLC) using a Phenomenex Luna C18 (250 × 21.2 mm, 100 Å, 10 µm) column (Supplementary Fig. 2). The appropriate fractions were pooled and lyophilized (purity > 98%) and analyzed using liquid chromatography-electrospray ionization-mass spectrometry (LC–ESI–MS) (Supplementary Fig. 2).

For testing the possible effects of ly-GnRH/CRZ peptide on feeding, locomotion, and egg-laying, 500 µg of synthetic peptide was first dissolved in 25 µL dimethylsulfoxide (DMSO) then diluted in 475 µL *Lymnaea* Ringer (50 mM NaCl, 2 mM KCl, 4 mM CaCl_2_, 4 mM MgCl_2_, 10 mM TRIS; pH = 7.5); hence, the given stock solution was 1 mg/mL with 5% DMSO. This was further diluted in *Lymnaea* Ringer to achieve the appropriate concentration (100 µg/mL with 0.5% DMSO) for injection. Relying on previous findings on *A. californica*^4^, specimens in the treated group were injected with 100 µL of this peptide solution directly into the hemocoel at the foot’s base using a needle (10 µg peptide/each animal). Specimens in the control group were injected with 100 µL *Lymnaea* Ringer with 0.05% DMSO.

### Behavioral assays

For behavioral assays, specimens were divided into control and ly-GnRH/CRZ injected experimental groups (n = 10 animals/group; n = 20 total animals/replicates). Experiments were performed in 3 independent series.

#### Feeding

Feeding behavior (number of rasps/2 min) was followed by placing the snails from the control and injected groups individually into a Petri dish filled with 90 mL low-copper artificial snail water (Supplementary Fig. 3). Specimens were food-deprived for two days before the test to be appropriately motivated. On the first observation day, after acclimatization for 10 min, 5 mL low-copper artificial snail water was added into the Petri dish and the number of rasps was counted for 2 min (i.e. spontaneous rasping in water). After this, 5 mL 20% sucrose solution, which evokes feeding activity, i.e. rhythmic opening/closing movements of the mouth^44^, was added into the Petri dish and the evoked feeding rate was counted for 2 min. On the second observation day, the specimens were injected with ly-GnRH/CRZ and the feeding activity was monitored two times (2 min and 2 h after injection). The feeding scores were obtained by subtracting the number of bites in response to water from the number performed in response to sucrose.

#### Locomotion

Locomotor activity was followed by placing the snails from the control and injected groups into experimental tanks (10 × 20 × 3 cm;^45^; Supplementary Fig. 4). Specimens were food-deprived for two days before the test to be appropriately motivated. On the first observation day (48 h before injection), after acclimatization for 10 min, the locomotion route of snails was marked continuously by a marker three times for 5 min. On the second observation day, the specimens were injected with ly-GnRH/CRZ, and locomotor activity was monitored three times in the first 45 min after injection. The locomotion test was also performed 24 h and 48 h after injection. Digital photographs of each animal were taken using a Nikon D5100 camera after the test. Based on individual pictures, the traces made by a single animal were measured (in cm) and analyzed with Mousotron v.8.2 software (BlackSun; www.techspot.com/download).

#### Egg-laying

Egg-laying behavior was monitored by placing the snails from the control and injected groups into holding tanks (with a 10 individuals/tank stocking density). Egg-laying was followed for five days after peptide injection (Supplementary Fig. 5). Each egg mass from the tanks was collected, the total number of laid egg masses and eggs was counted and the quality of egg masses was evaluated using a Leica M205c stereomicroscope equipped with a DFC3000G (Leica) digital camera. Such parameters as too much gelatinous material, oocytes out of the eggs, or the presence of empty eggs, which could be interpreted as a negative indication regarding damaged reproduction of the snails, were not observed during the test.

### Retrograde labeling by nickel-lysine backfill

For nickel-lysine backfill of the ventral- and anterior lobe of the right cerebral ganglion, the whole CNS with the penis nerve was dissected from snails (n = 5) and pinned out on a Sylgard-coated dish containing *Lymnaea* Ringer. Penis nerve was trimmed to a length of about 1–1.5 cm and the cut end shocked osmotically in distilled water for 20 s. The cut end was then placed in a chamber containing a nickel–lysine solution (0.38 M NiSO_4_ × 6H_2_O and 1.2 M L-lysine, #L5501, Sigma-Aldrich). The whole CNS was incubated overnight at room temperature. After that, nickel was precipitated by adding 5–10 drops of a saturated alcohol solution of rubeanic acid (dithiooxamide, #STBB5837, Sigma-Aldrich) and incubating for 30 min at room temperature (Supplementary Fig. 6). Afterward, samples were processed for IHC described below (IHC section).

### IHC

The heart and penial complex (comprising the preputium, penis, and retractor muscles^17,36^; Supplementary Fig. 7) were dissected from individual snails. These tissues and the backfilled whole CNS samples described above were pinned out on a Sylgard-coated dish containing 4% paraformaldehyde in 0.1 M phosphate-buffered saline (pH = 7.4) overnight at 4 °C. The procedure followed a protocol established previously^5^. In the case of heart and penial complex, besides the GnRH/CRZ antiserum (#AS203-2, EZbiolab; antigen was a synthetic undecapeptide: CNYHFSNGWYA-amide)^4^, we also used a mouse anti-serotonin (5-HT) antiserum (#Mo75801-2, Dako, Glostrup, Denmark) diluted 1:500 which had been used successfully several times in *L. stagnalis* research^46–49^.

### Statistical analysis

Statistical analysis was carried out using OriginPro8 2018 software (OriginLab Corp., Northampton, Massachusetts, USA). The normality of the dataset was investigated using the Kolmogorov–Smirnov test, homogeneity of variances between groups was investigated using the F-test. In the case of feeding activity and egg-laying behavior, differences among groups were analyzed by a two sample t-test. For locomotor activity, two-way repeated-measures ANOVA was used to assess the main effects of time, treatment, and time x treatment interaction. This analysis was followed by a two sample t-test to identify significant differences between the groups within a given time point. Differences were considered statistically significant at *P* < 0.05 (*), *P* < 0.01 (**), and *P* < 0.001 (***).

## Supplementary Information

Supplementary Information.

## Data Availability

The datasets generated during and/or analyzed during the current study are available from the corresponding author on reasonable request.
