# Supplementary material for: Functional characterization and related evolutionary implications of invertebrate gonadotropin-releasing hormone/corazonin in a well-established model species
Source: Sci Rep. 2021 May 11;11:10028. doi: 10.1038/s41598-021-89614-5 (PMC8113230; doi:10.1038/s41598-021-89614-5)
Supplement: Supplementary file 1 — Supplementary Information. [file 41598_2021_89614_MOESM1_ESM.docx]

**Supplementary information**

**Functional characterization and related evolutionary implications of invertebrate gonadotropin-releasing hormone/corazonin in a well-established model species.**

^1^István Fodor, ^1^Réka Svigruha, ^2^Zsolt Bozsó, ^2^Gábor K. Tóth, ^3^Tomohiro Osugi, ^3^Tatsuya Yamamoto, ^3^Honoo Satake, and ^1^Zsolt Pirger*

^1^NAP Adaptive Neuroethology, Balaton Limnological Research Institute, Balaton Limnological Research Institute, Eötvös Loránd Research Network (ELKH), Klebelsberg Kuno u. 3., H-8237 Tihany, Hungary

^2^Department of Medical Chemistry, University of Szeged, Szeged, Hungary

^3^Bioorganic Research Institute, Suntory Foundation for Life Sciences, 8-1-1 Seikadai, Seika, Souraku, Kyoto 619-0284, Japan

*****Corresponding author; E-mail: [pirger.zsolt@blki.hu](mailto:pirger.zsolt@blki.hu) (Z Pirger)

**Supplementary Table 1**. List of identified molluscan GnRH/CRZ molecules.

| **Species** | **Accession number** | **Reference** |
| --- | --- | --- |
| common octopus (*Octopus vulgaris*) | AB037165 | Iwakoshi et al., 2002 |
| sea hare (*Aplysia californica*) | NM_001204553 | Zhang et al., 2008 |
| owl limpet (*Lottia gigantean*) | FC805608 | Tsai and Zhang, 2008 |
| swordtip squid (*Loligo edulis*) | FC805608 | Onitsuka et al., 2009 |
| common cuttlefish (*Sepia officinalis*) | precursor identified by cDNA cloning | Di Cristo et al., 2009 |
| yesso scallop (*Patinopecten yessoensis*) | AB486004 | Treen et al., 2012 |
| pacific oyster (*Crassostrea gigas*) | HQ712119 | Treen et al., 2012 |
| mediterranean limpet (*Patella caerulea*) | precursor identified by cDNA cloning | De Lisa et al., 2013 |
| manila clam (*Ruditapes philippinarum*) | KF891317 | Song et al., 2015 |
| ass's-ear abalone (*Haliotis asinina*) | KP719130 | Nuurai et al., 2015 |
| smooth Australian abalone (*Haliotis laevigata*) | KP719129 | Nuurai et al., 2015 |
| marsh snail (*Biomphalaria glabrata*) | precursor *in silico* identified in the transcriptome data | Adema et al., 2017 |
| disk abalone (*Haliotis discus hannai*) | MK089558 | Kim et al., 2017 |
| the great pond snail (*Lymnaea stagnalis*) | MN385595 | Fodor et al., 2020 |

**Supplementary Figure 1**. HPLC profile of the retained material on a reverse-phase column (ODS-80Ts). The retained material loaded onto the column was eluted with a linear gradient of 4.5-49.5% ACN in 0.1% TFA at a flow rate of 1 mL/min for 50 min and collected every 1 min.


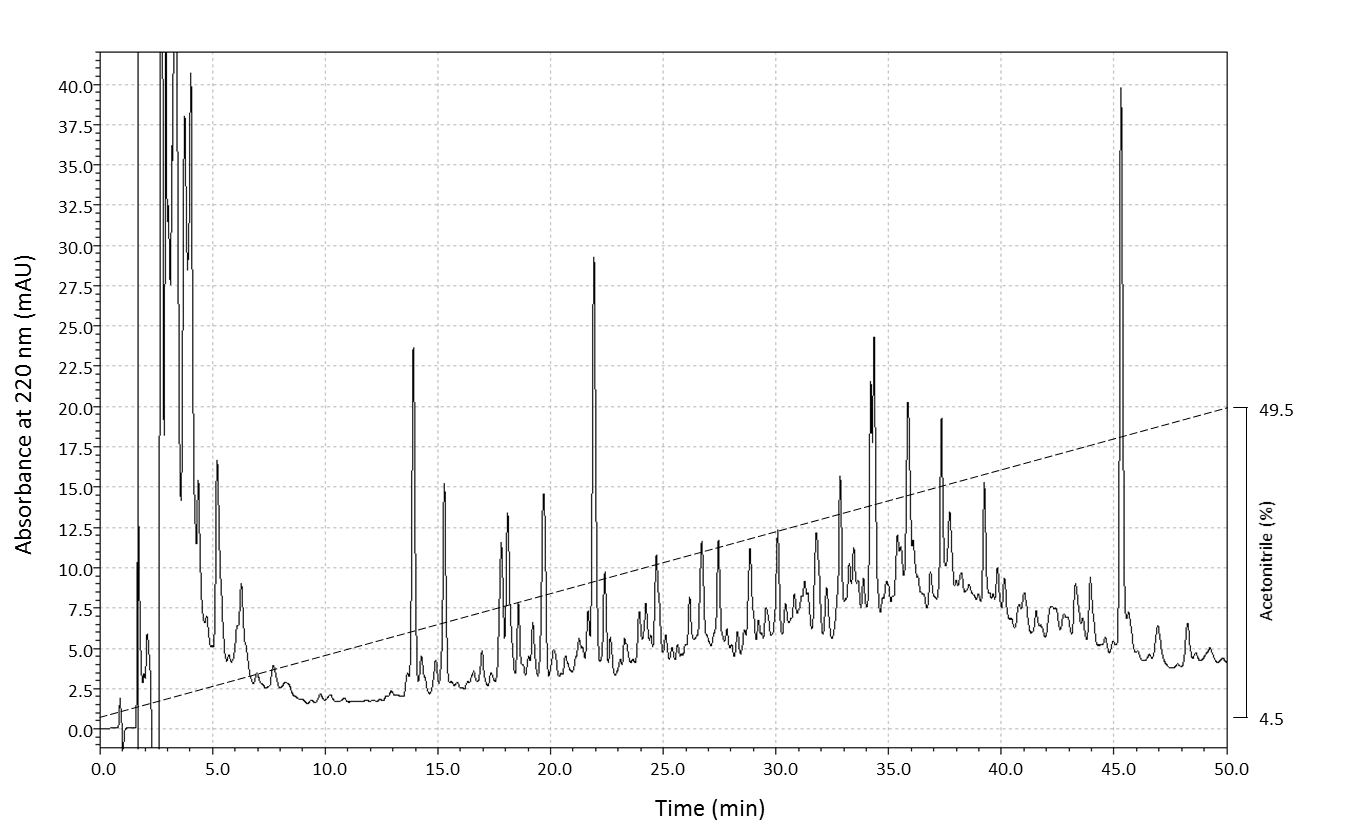


**Supplementary Figure 2**. HPLC trace of the crude (**A**) and purified (**B**) synthetic active peptide as well as MS spectrum of the purified peptide (**C**). Solvent A: 0.1% TFA in water; Solvent B: 0.1% TFA/ 80% acetonitrile/water; Gradient: 25-45% Solvent B in Solvent A over 20 minutes

**(A)**

**
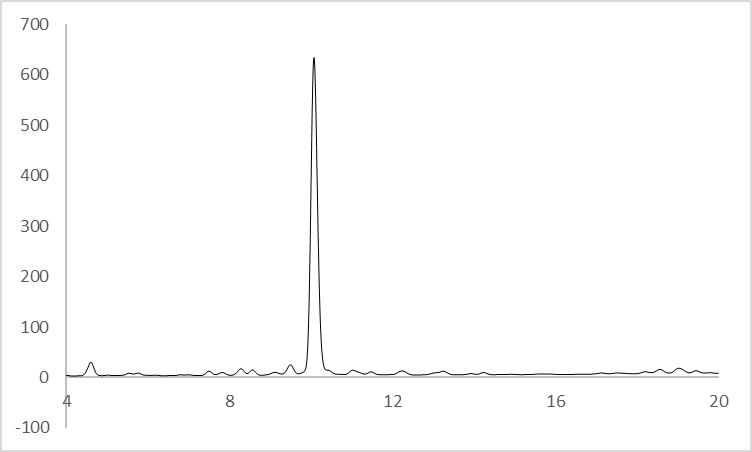
**

**(B)**


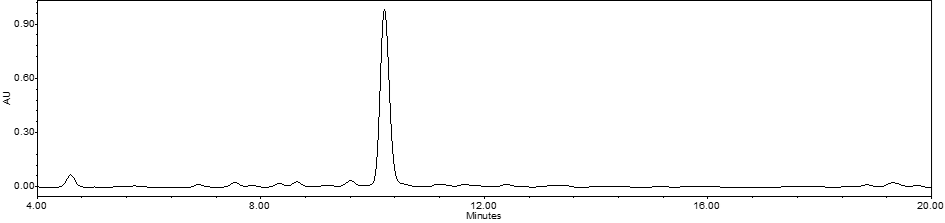


**(C)**


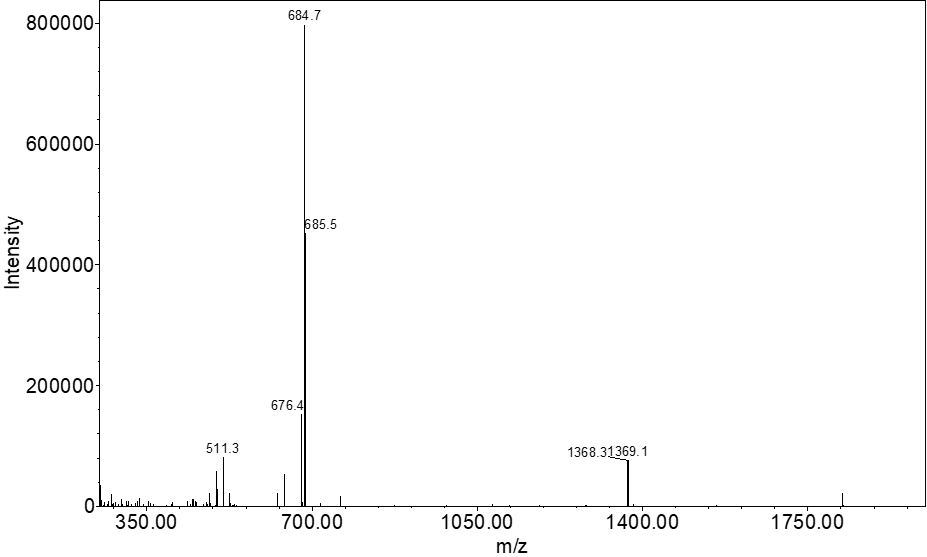


**Supplementary Figure 3**. Monitoring of feeding activity. (**A**) First observation day: characterization of feeding activity 24 h before ly-GnRH/CRZ injection. Second observation day: characterization of feeding activity after ly-GnRH/CRZ injection. (**B**) Experimental arrangement and rhythmic opening/closing movements of the mouth.


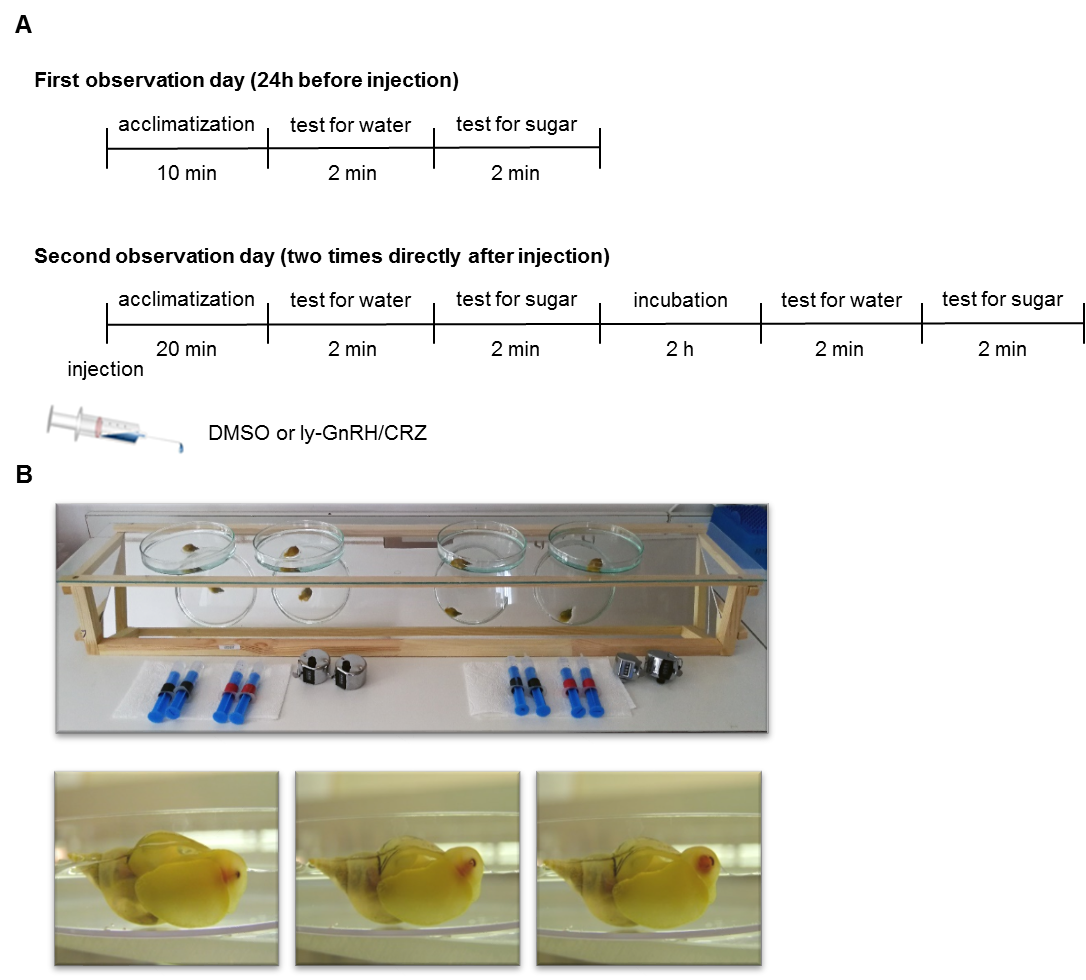


**Supplementary Figure 4**. Monitoring of locomotor activity. (**A**) First observation day: characterization of locomotor activity 48 h before ly-GnRH/CRZ injection. Second and third observation days: characterization of locomotor activity after ly-GnRH/CRZ injection. (**B**) Experimental arrangement and the locomotion route of snails.


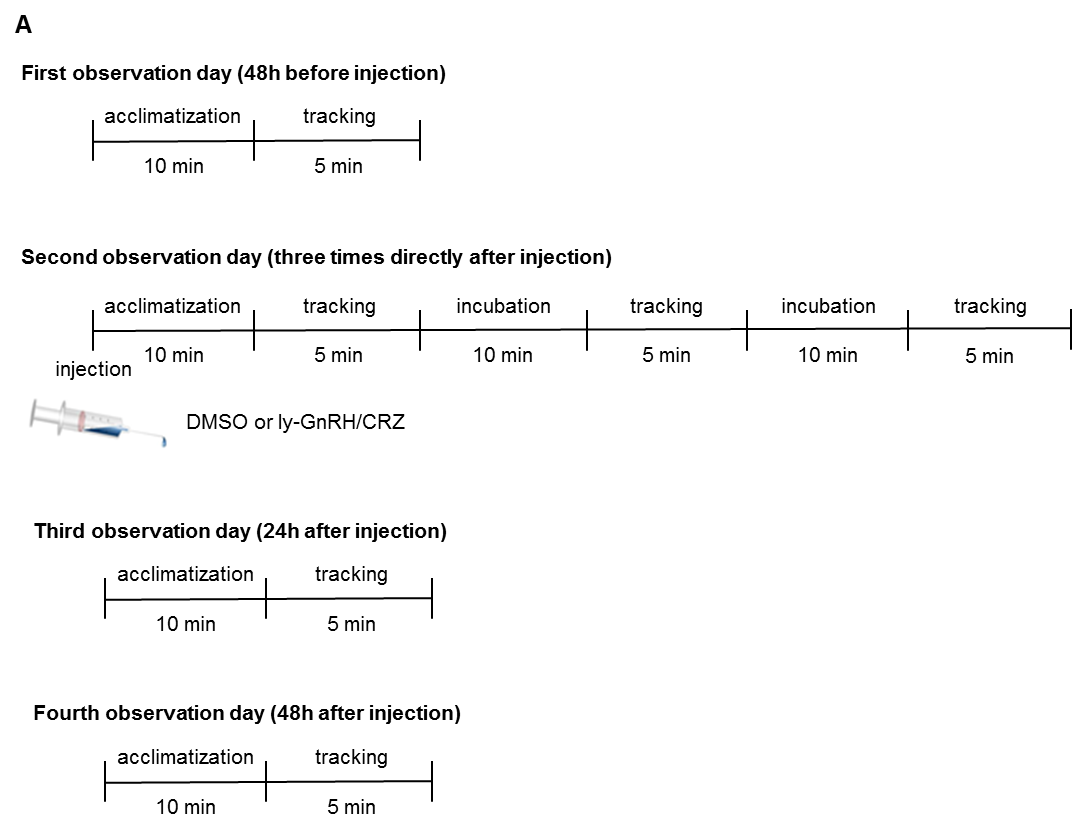


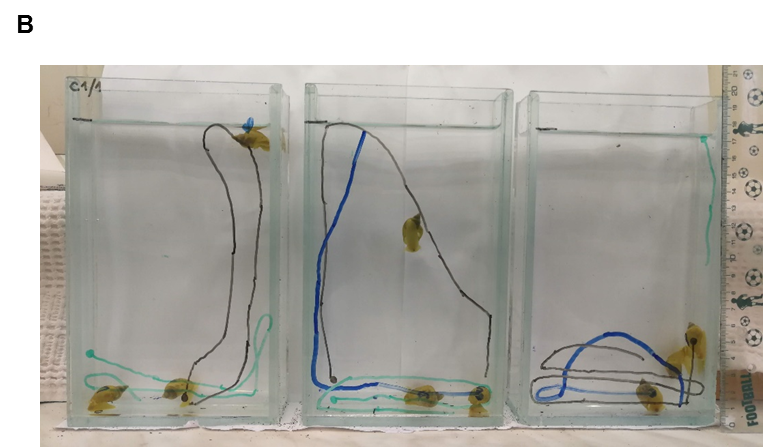


**Supplementary Figure 5**. Monitoring of egg-laying. (**A**) First observation day: characterization of egg-laying behavior for 5 days after ly-GnRH/CRZ injection. (**B**) Egg mass containing normal (asterisk) and polyembryonic eggs (arrows).


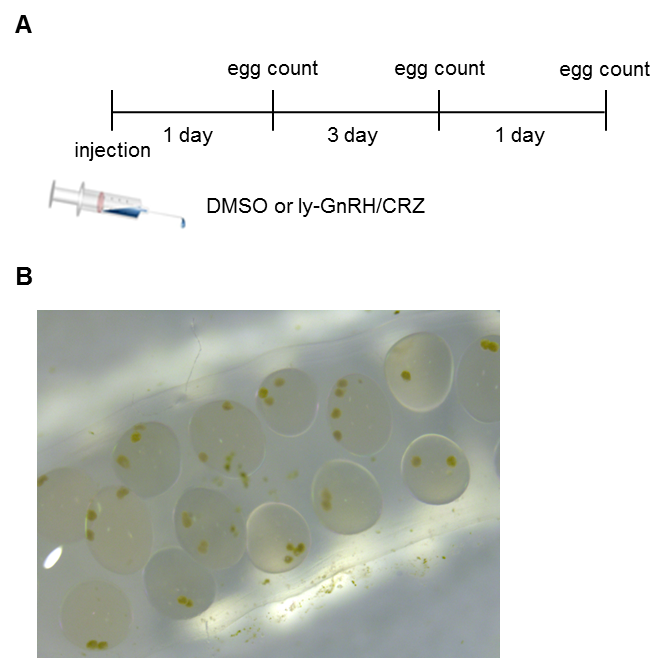


*

**Supplementary Figure 6**. Retrograde labeling from the penis nerve (pen) by nickel backfill. Penis nerve was placed in a chamber containing a nickel-lysine solution. After overnight incubation, nickel was precipitated by rubeanic acid (intensification). Dashed square on the left panel indicates the section seen on the right panel. Coinciding with the literature (De Boer et al., 1997), neurons of the anterior lobe (al) and ventral lobe (vl) of the right cerebral ganglion (CeG), the pedal Ib cluster (Ib) of the right pedal ganglion (PeG), and dispersed neurons from the right pleural ganglion (PlG) and the right parietal ganglion (not shown) were stained. Abbreviations: CNS – central nervous system; cpec – cerebro-pedal connective; cc – cerebral commissure; db – dorsal body


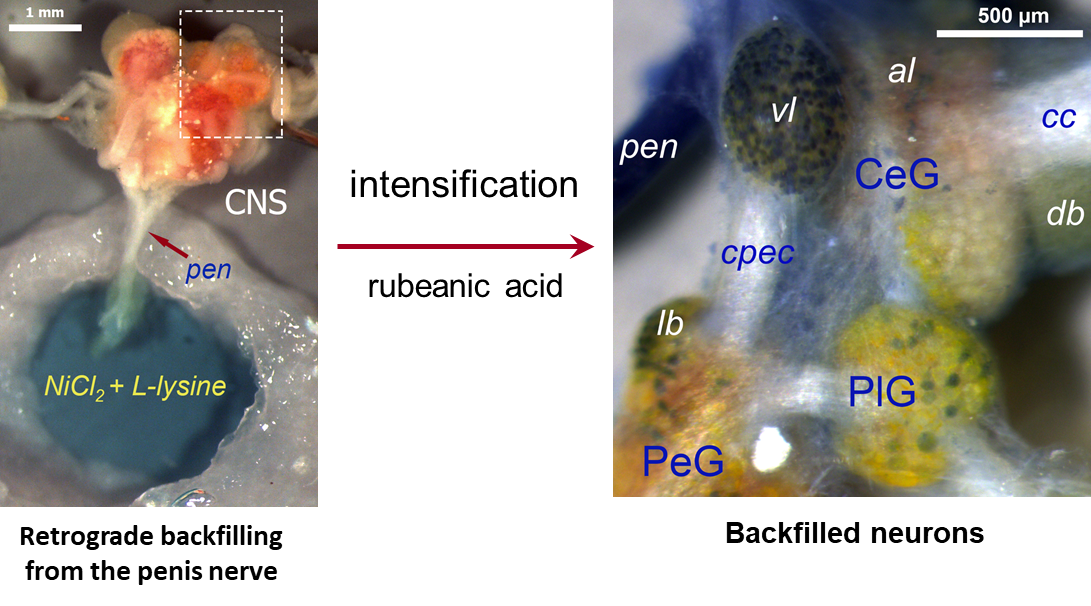


**Supplementary Figure 7.** (**A**) Morphology of vas deferens and penial complex (comprising the preputium, penis, and retractor muscles [indicated by numbers]). (**B**) Representative hematoxylin and eosin stain of preputium. Abbreviations: LM – longitudinal muscle; E – epithelium. Scale bars = 2.5 mm (A) and 10 µm (B)

A

**
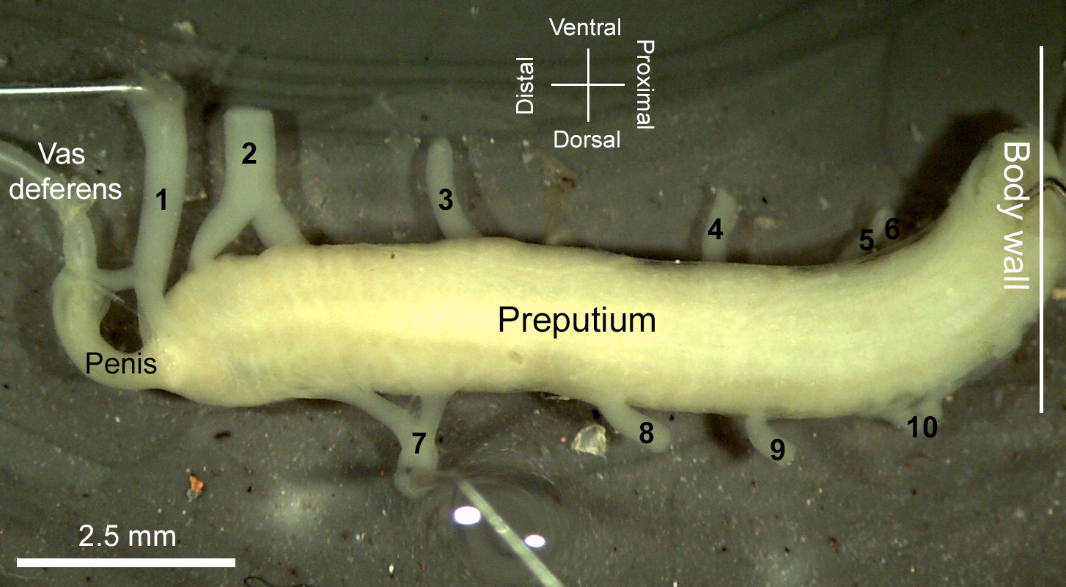
**

**
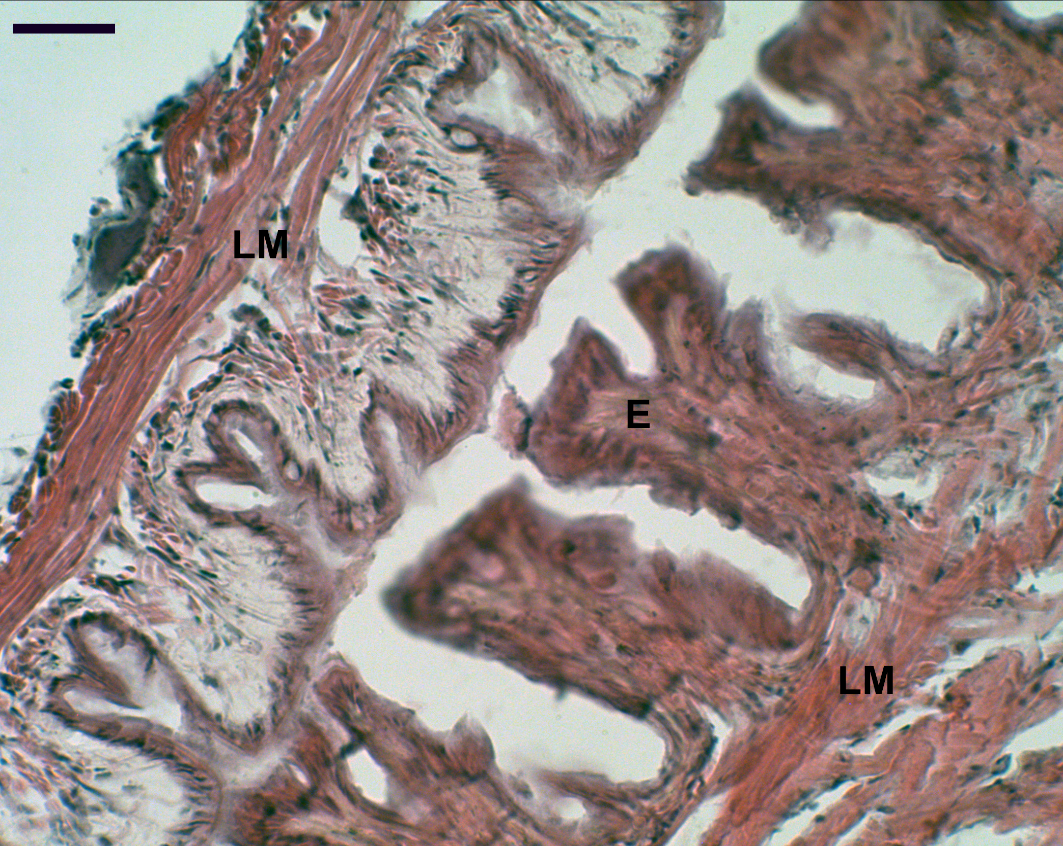
**

B
